# Behavioral problems of school children: impact of social vulnerability, chronic adversity, and maternal depression

**DOI:** 10.1186/s41155-018-0089-9

**Published:** 2018-06-04

**Authors:** Ana Karina Braguim Martineli, Fernanda Aguiar Pizeta, Sonia Regina Loureiro

**Affiliations:** Universidade de São Paulo (USP), Rua Tenente Catão Roxo, 2650, Ribeirão Preto, SP CEP: 14051-140 Brazil

**Keywords:** Behavior, School children, Adversities, Maternal depression, Social vulnerability

## Abstract

This study’s objective was to identify the predictive effect of indicators concerning social vulnerability, chronic adversity, and maternal depression on behavioral problems among school-aged children, according to the perceptions of mothers and teachers, considering the presence or absence of difficulties in the contexts of family and school. A total of 85 pairs of mothers and school children were distributed into three groups according to the behavioral problems identified. A General Questionnaire, the PHQ-9, the Chronic Adversity Scale, and the (Strengths and Difficulties Questionnaire) SDQ were applied to the mothers; the Raven’s Colored Progressive Matrices were applied to the children; and the SDQ was applied to the teachers. Data were analyzed with descriptive, predictive, and comparative statistical procedures (*p* ≤ 0.05). The results reveal the presence of cumulative risks for children with behavioral problems; mothers more frequently identified behavioral problems than teachers; and maternal depression was a predictor for behavioral problems. Such findings are relevant for devising mental health programs.

## Background

The behavior of school-aged children is an important indicator of adaptation in this stage of development; however, not all children are successful and may present externalizing or internalizing behavioral problems, expressed within the family and/or school contexts (Achenbach, Ivanova, Rescorla, Turner, & Althoff, 2016; Linhares & Martins, 2015; Marturano, 2013).

Families present structural and internal dynamics that may contribute to either risk or protective outcomes in terms of child development (Macana & Comim, 2015; Walsh, 2016) so that adaptive difficulties associated with behavioral problems are frequent among children (Pizato, Marturano, & Fontaine, 2014). Considering the relevance of the influence exerted by the family context on school children, various studies have addressed the association of cumulative adversity present in the family context and the outcomes of behavioral problems among children (Duncombe, Havighurst, Holland, & Frankling, 2012; Leis, Heron, Stuart, & Mendelson, 2014; Pizeta, Silva, Cartafina, & Loureiro, 2013).

When analyzing the potential risk factors for the development of psychopathologies in 252 children and adolescents who are victims of domestic violence, Hildebrand, Celeri, Morcillo, and Zanolli (2015) verified that 92.8% of the participants were exposed to at least one risk factor. The authors also noticed that the association of two or more risk factors were present in 53.2% of the sample, namely family conflicts, mental health problems within the family, gender violence between parents, family involvement with drug trafficking and criminal behavior, and the abusive consumption of alcohol by parents or legal guardians, among others.

Therefore, among the events listed as conditions that predispose children to behavioral problems, we highlight indicators of chronic adversity and mental health conditions affecting the parents, especially maternal depression, as risk conditions acknowledged to have a negative impact on children. Additionally, the presence of variables related to social vulnerability is also identified based on conditions or events of life that may interfere in the course of developmental outcomes for children, contributing to the maladaptation of children in typical developmental tasks when experiencing risk conditions.

Considering social vulnerability in the population in general, low socioeconomic status and unemployment among mothers were identified as predictors of behavioral problems among children in situations of poverty, according to a study conducted by Bele, Bodhare, Valsangkar, and Saraf (2013) of children in India. In the Brazilian context, Correia, Saur, and Loureiro (2014) conducted a cohort study and identified an association of behavioral problems with low socioeconomic status for boys and low maternal education and larger families for girls. In the same direction, Pizato et al. (2014) verified association between improved socioeconomic conditions with fewer behavioral problems and more social skills in school-aged children; Saur and Loureiro (2015) identified associations between behavioral problems among 10-year-old children with low maternal educational level and low socioeconomic status and families with more than four members. It is also considered that the family socioeconomic condition can influence the cognitive performance of children, being this condition strongly related to other environmental aspects such as maternal depression (Piccolo et al., 2012).

In regard to the parents’ mental health, maternal depression, especially given its high prevalence and recurrence (World Health Organization [WHO], 2017a, 2017b), stands out as a form of adversity in different periods of child development, impacting the behavior of school-aged children (Bagner, Pettit, Lewinsohn, & Seeley, 2010; Callender, Olson, Choe, & Sameroff, 2012; Edwards & Hans, 2015; Loosli, Pizeta, & Loureiro, 2016). Such a psychopathology, however, is associated with other adverse contextual conditions, favoring cumulative risk in the family context (Kessler, 2012). Note that the condition of cumulative risk has been acknowledged in the literature as having a greater impact for outcomes among children compared to the presence of a single risk (Evans, Li, & Whipple, 2013). Thus, this justifies the relevance of studying potential associations between maternal depression and behavioral problems, including other variables in the family environment, as proposed in this paper.

Indicators of social vulnerability and clinical characteristics of maternal depression have been identified as relevant factors to understanding risk conditions for child development. Barker, Copeland, Maughan, Jaffee, and Uher (2012) monitored children from their first year of life up to the age of 7 and verified that, in comparison to children of mothers without depression, children of mothers with depression were more frequently exposed to 10 out of the 11 risk factors assessed in the study, among which, low socioeconomic status, single parent, physical abuse, low maternal education, and drug and alcohol consumption. The frequency of exposure was at a significant level. Indicators concerning the severity of depression and anxiety were examined by Leis et al. (2014), in a sample of 2891 mother-child pairs, taking into account the perspectives of mothers and teachers. The authors found an association between severe depressive symptoms during pregnancy and more frequent behavioral problems at the age of 10 and 11 years old, according to the reports of teachers. Conners-Burrow et al. (2016), who took into account the assessment of mothers, determined that early contact with maternal mild depressive symptoms increased the risk of children presenting internalizing and externalizing behavioral problems during school-age years.

Still considering chronic risk and adversity and their influence on child behavior, we highlight the study by Wang, Christ, Mills-Koonce, Garrett-Peters, and Cox (2013), who found associations between externalizing problems among 4- to 12-year-old children and the use of more rigid control and low maternal educational levels. The study by Bouvette-Turcot et al. (2017) reports that exposure to more adversity and low family income during childhood was associated with the development of depressive symptoms in adulthood.

When addressing behavioral problems, one issue that arises refers to the source of assessments, considering that children and adolescents may present problems in a specific context but not in another, for instance, family versus school, indicating a need to obtain assessments from multiple informants, especially from parents or legal guardians and teachers (Martoni, Trevisan, Dias, & Seabra, 2016; Miller, Martinez, Shumka, & Baker, 2014). In this direction, some studies draw attention to the low to moderate level of agreement obtained between informants and to the relevance of such information to implementing clinical practices intended to address specific contexts in which children present problems (De Los Reyes et al., 2015; Martel, Markon, & Smith, 2017). Despite disagreement among the various informants, different observers provide different perspectives of the same problem (Miller et al., 2014). Each observer, though, can provide potentially valuable data in regard to the same patient (De Los Reyes, Thomas, Goodman, & Kundey, 2013; Clark, Durbin, Hicks, Iacono, & McGue, 2017), taking into consideration different contexts.

With school-aged children in mind, mothers and teachers have a privileged opportunity to observe the behavior of children, since the family and school are the primary contexts of development where competence in specific tasks inherent to this period is acquired (Achenbach et al., 2008), as previously mentioned. Some studies addressing the behavior of children according to the assessments of parents and teachers highlight the discrepancy between such assessments. Johnson, Hollis, Marlow, Simms, and Wolke (2014) used the Strengths and Difficulties Questionnaire (SDQ) to assess 219 children aged 11 years old who were born prematurely. The authors verified that the parents considered their children to present more emotional, attention, and relationship problems compared to the assessments provided by teachers. The informants agreed only in regard to the assessment of problems related to hyperactivity, which indicates the importance of using combined assessments. Kovess et al. (2015) conducted a study with 9084 children between 6 and 9 years of age, from seven countries (Italy, the Netherlands, Germany, Romania, Bulgaria, Lithuania, and Turkey), in which both teachers and parents were informants. The objective was to identify risks to the mental health of students. They verified that the teachers found the children to present more externalizing problems and fewer internalizing problems when compared to the parents’ assessments.

Even though assessments provided by multiple informants are considered relevant, the literature still lacks data. This study seeks to fill this gap and is intended to produce new data concerning the behavior of school children assessed by mothers and teachers, considering conditions in which children live with maternal depression and other adversities. Therefore, this study is intended to fill the gaps pointed out by De Los Reyes et al. (2015) concerning the need for further research using the assessments of multiple informants and addressing the specifics of contexts in which behavioral problems manifest, as a way to improve understanding regarding such problems, focusing on maternal depression. According to Goodman et al. (2011), there is a need for studies focusing on the multiple adversities presented in the family environment, taking into account the influence of maternal mental health when assessing the behavior of children, as indicated by Leis et al. (2014).

Therefore, the objective was to identify the behavioral profile of school children and associations between the evaluation of mothers and teachers, identifying the level of agreement among the informants. In addition, we aimed to evaluate the predictive effect of indicators concerning social vulnerability, chronic adversity, and maternal depression on behavioral problems presented by school children, according to the perspectives of mothers and teachers, considering the presence or absence of difficulties in both family and school contexts. The hypothesis guiding this study was that social vulnerability, chronic adversity, and maternal depression predict more frequent behavioral problems among school children in both developmental contexts, family and school, assessed by mothers and teachers, respectively.

## Methods

A cross-sectional, correlational, predictive, comparative design was adopted using data obtained with different techniques from different sources, namely mothers, teachers, and children. The study was approved by the Institutional Review Board (no. 36415514.5.0000.5407) and complied with the ethical recommendations proposed by the Declaration of Helsinki.

### Participants

A total of 85 mother-child pairs and 16 teachers from a public school located in the state of São Paulo, Brazil, took part in this study. The participants were assigned to three groups, according to the children’s indicators of behavioral problems assessed by their mothers and teachers, namely G1 = 18 children with behavioral problems according to their mothers and teachers, G2 = 39 children with behavioral problems according to their mothers or teachers, and G3 = 28 children without behavioral problems according to their mothers and teachers.

According to the inclusion criteria, mothers were aged between 25 and 45 years old, 34.5 years old on average (SD = 5.51), and all were literate. The children were aged between 7 and 10 years old, 8.8 years old on average (SD = 1.06) and were homogeneously distributed into three groups. In regard to the children’s sex, 39 were girls and 46 were boys, making a balanced distribution according to sex impossible: G1 presented significantly more boys than girls when compared to the G2 and G3 (*p* = 0.05; *p* = 0.02, respectively). In order to assess the weight of this variable for the presence or absence of behavioral problems among children, as assessed by both their mothers and their teachers, an ordinal regression analysis was performed considering the sex of the children, which revealed a model that did not present the minimum criteria regarding slope homogeneity [chi-square (1) = 5.285; *p* = 0.022; *D*(1) = 5.524; *p* = 0.019], that is, it is not a model that fits data under analysis.

The inclusion criteria are that the children live with their biological mothers, rather than adoptive mothers, and have attended at least 1 year of primary school. Institutionalized children or those with apparent physical or mental disabilities were excluded. The assessment of children was initiated after consent was obtained from their mothers, and only one child per family was included in the study. In regard to the teachers, only those who had had at least 3 months of contact with the children and taught the children whose mothers explicitly consented to the assessment of their children at school were included. In accordance with the principles of good research practices, the participation of mothers and teachers was voluntary, without incentive payment mechanisms that stimulated the involvement with the research. A lecture was offered to the school on the behavior and learning of school children.

### Instruments

#### Raven’s Colored Progressive Matrices (Raven)

The Raven is an instrument standardized by Angelini, Alves, Custódio, Duarte, and Duarte (1999), to assess the intellectual level of Brazilian children between 5 and 11 years old. It is a psychological test of non-verbal intelligence; the objective of which is to assess one’s analogical reasoning as a general factor, composed of three series: A, AB, and B, each with 12 problems. It presents good psychometric qualities, inferred by construct validity, internal consistency, with item-total correlation between 0.30 and 0.80 for most items, as well as precision, inter-item coefficient of correlation for the total sample equal to 0.92 (Angelini et al., 1999). Children presenting potential cognitive deficits, who presented percentiles lower than 25, were excluded from the study (Muniz, Gomes, & Pasian, 2016), balancing groups according to the percentiles obtained by the children.

#### Patient Health Questionnaire-9 (PHQ-9)

The PHQ-9 is a module directly based on the diagnostic criteria for major depression disorder from the DSM-IV, proposed and validated by Spitzer, Kroenke, and Williams (1999) and by Kroenke, Spitzer, and Williams (2001). The questionnaire enables both screening for signs and symptoms of current major depression, as well as classifying levels of severity, from mild to moderate or severe; the greater the score, the more indicators of problems the individual presents. It is composed of nine items assessed by an ordinal scale that measures the frequency of signs and symptoms of depression in the last 2 weeks. According to the instrument’s technical instructions, the total score was used so that scores greater than or equal to 10 indicate the presence of depressive symptoms, while scores lower than 10 indicate an absence of such symptoms. The Brazilian version used in this study was translated by *Pfizer* *(Copyright* © 2005 *Pfizer* Inc., New York, NY), the reliability of which was verified by Osório, Mendes, Crippa, and Loureiro (2009), presenting satisfactory psychometric indicators.

#### Strengths and Difficulties Questionnaire (SDQ)

The SDQ was developed by Goodman (1997) and is intended to assess the behavior of children and adolescents, aged between 4 and 16 years old, by screening their behavioral strengths and difficulties. There is a version for children and adolescents between 11 and 16 years of age, a version for parents, and another for teachers. The SDQ is composed of 25 items subdivided into five subscales: emotional symptoms, conduct problems, hyperactivity, peer relationship problems, and pro-social behavior, with five items each. It provides raw scores and cutoff points for each of the subscales, as well as a total score for difficulty that is obtained by totaling the four behavioral problem scales. Scores are classified as normal, borderline, and abnormal. It was translated to Portuguese and adapted for Brazilian sociocultural characteristics by Fleitlich, Cortázar, and Goodman (2000), while psychometric data, concerning validity and reliability, were described by Woerner et al. (2004), presenting good indicators. In this study, based on individual scores and cutoff points established for the Brazilian population, we considered the outcome variable for children classified as normal or borderline, according to the SDQ, to be “without difficulties,” while those who were classified as abnormal to be “with difficulties.” These outcomes were grouped with the assessments performed by the mothers and teachers, according to the distribution in the groups.

#### Chronic Adversity Scale (CAS)

The CAS was proposed by Marturano (1999) and is intended to identify recurrent adverse events that may have taken place in a child’s life and happened repeated times or lasted 1 year or longer. It is composed of 18 items addressing issues concerning chronic adversity regarding the child’s or the parents’ health, parents’ temperament, and potential family or marital conflicts. The scale is completed by the mothers based on a list of adverse conditions that may have developed in the lives of children since birth, specifying the duration in years and the child’s period of life at the time. Each item is scored either 0 (absence of recurrence or chronic nature of the event in the child’s life) or 1 (the event was recurrent or has a chronic nature); the sum of all 18 items results in the total score, which is used to identify the existence of chronic events.

#### General Questionnaire

This questionnaire addresses sociodemographic data and specific information concerning the mothers’ age, marital status, and educational level; the families’ monthly income and socioeconomic status; and the age, sex, and education of the children included in the study. The items from the Brazil Economic Classification Criteria, developed by the Brazilian Association of Survey Companies (2015), were used to assess socioeconomic conditions. Such information was used to characterize the participants and groups, as well as to identify social vulnerability indicators, including low maternal and paternal education, single-parent families, low socioeconomic status, and low family income, as well as being recipients of governmental financial support.

### Data collection procedures

Preferably, data were collected at school in a private room, or in the families’ homes when requested by the mothers, in which case we sought to preserve the respondents’ privacy and convenience. All interviews were held by the first researcher, who is a psychologist and properly trained in the application of instruments.

Initially, 427 families received an invitation letter, which was delivered to the children in their classrooms. The 260 families who responded to the invitation were contacted by phone with the objective to provide clarification about the study’s objectives and schedule an assessment. A total of 154 families accepted the invitation to cooperate with the study, but nine of these were excluded because the grandmothers were the primary caregivers of these families’ children. Of the 145 mothers scheduled for assessment, 43 did not attend the interviews, resulting in 102 families. Seventeen of these did not meet the inclusion criteria: adolescent mothers or mothers older than 45 years of age, children exclusively living with their fathers, and children with characteristics that were not homogeneous with those presented by the groups. Thus, a total of 85 mother-child pairs were included and assessed.

Of the 427 families initially invited to participate in this research, 316 refused to collaborate with the survey and 26 were excluded because they did not meet the inclusion criteria.

The instruments were individually and in-person applied to mothers in a single section according to the following order: General Questionnaire, PHQ-9, CAS, and SDQ, with an average duration of 60 min. The researcher read the instruments and checked the responses while the mothers had a copy of the instruments to accompany the reading. This procedure was adopted to deal with potential difficulties or fatigue that the reading could produce in the mothers, given their level of education or potential depressive symptoms, though the mothers presented a minimum level of literacy that enabled them to understand the questions posed by the instruments.

The children were assessed at school in individual sessions that lasted an average of 15 min. After briefly establishing rapport, the Raven’s Colored Progressive Matrices was applied. The three groups were compared according to the percentiles children obtained in order to balance the groups in regard to this variable. Note that there were no significant statistical differences between them in regard to the children’s cognitive performances (G1: $\overline{x}$ = 75.1; *σ* = 19.12; G2: $\overline{x}$ = 76.0; *σ* = 13.44; G3: $\overline{x}$ = 66.9; *σ* = 14.97).

The 16 teachers collectively completed the SDQ, focusing on the behavior of 85 children who had been previously assessed by their mothers, at the regular time scheduled for a meeting concerning collective teaching work. Each teacher was supposed to assess up to five students per meeting, with an average duration of 50 min and approximately 10 min per child. The teachers filled in the questionnaire, and the researcher remained in the room during the assessment to clarify potential doubts.

### Data treatment and analysis

The PHQ-9, Raven, SDQ, and CAS were coded according to the purpose of each instrument. The assessments concerning the behaviors of children performed by the mothers and teachers using the SDQ were used as distinct sources in paired samples, in order to assign the participants to one of the three groups.

Coded data were typed in an Excel® spreadsheet and checked by independent reviewers. The statistical analyses were performed using IBM SPSS Statistics (v. 23; IBM SPSS, Chicago, IL), and a significance level of 0.05 was adopted.

The reliability of the PHQ-9 was verified for this sample using Cronbach’s alpha, which presented good psychometric quality (*α* = 0.87). The reliability of the SDQ (*n* = 85) for the totality of items regarding difficulties was based on the mothers’ (*α* = 0.77) and teachers’ answers (*α* = 0.89), as well as items of the pro-social behavior scale answered by mothers (*α* = 0.71) and teachers.

Normality tests (Kolmogorov-Smirnov, with Lilliefors significance correlation, and Shapiro-Wilk) were performed to guide decision-making regarding the statistical tests used for each set of variables.

The behavioral profile of children, obtained through the assessments of mothers and teachers and represented by the raw scores obtained on the scales addressing problems and pro-social resources and the total scale of difficulties in the SDQ, was analyzed using descriptive and comparative statistics by means of the Wilcoxon test. Indicators of the presence and absence of behavioral problems among children, assessed by the mothers and teachers using the SDQ, were compared using the McNemar test. Inter-observer agreement was also verified using the Kappa coefficient, while the criterion proposed by Landis and Koch (1977) was adopted.

The sociodemographic data and profiles of social vulnerability and chronic adversity were analyzed using descriptive statistics, while the groups were compared using chi-square and Kruskal-Wallis tests. After the univariate analysis, the predictive effect of cumulative adverse conditions on school children’s behavioral problems was assessed using ordinal regression analysis (Maroco, 2014), adopting criteria proposed by Field (2013) for the inclusion of predictive variables.

The weight of contextual adverse cumulative variables for the children’s behavioral problems was tested using ordinal regression analysis based on the significant variables identified in the comparison between groups. The predictive variables were included in the model (family income, mother’s education, and maternal depression were included as factors, and the score of chronic adversity was included as a covariant), independently tested, and combined with the sex of children, because the distribution of children in the groups according to sex was not homogeneous. Additionally, the contextual variables of cumulative risk were jointly tested in a multivariate model.

The analysis of the social vulnerability indicators (income and maternal education) and maternal depression, in one analysis disregarding the sex of children and then one considering the sex of children, showed that the models did not fit the data. The analysis of the models that included chronic adversity, as a single variable or associated with the sex of children, revealed statistically significant models, with very small effect sizes, in which independent variables did not predict the behavioral outcome among children, thus did not present relevant results. Afterwards, the multivariate model including maternal depression, social vulnerability indicators, and chronic adversity was tested and presented goodness of fit and is the model presented here.

## Results

Based on the objectives proposed, the results are presented taking into account the analyses concerning the children’s behavioral profiles according to the assessments of mothers and teachers as distinct sources, comparisons between G1, G2, and G3 regarding profile of social vulnerability, maternal depression, and chronic adversity, as well as the predictive effect of significant variables on the children’s behavioral problems assessed by mothers and professors, as combined sources of information.

### The children’s behavioral profiles

Table 1 presents the behavioral profiles of the children assessed, according to the SDQ, by mothers and teachers as two different sources, adopting the presence or absence of behavioral problems verified by the SDQ and total difficulties as the outcome of the development of school children.

**Table 1:** Comparisons regarding the behavioral profile of children (SDQ—mothers and teachers) (*n* = 85)

Respondents SDQ	Mothers	Teachers	*p* value*
	*f* (%)	*f* (%)	
Emotional symptoms			
With difficulties	52 (61)	05 (06)	< 0.001
Without difficulties	33 (39)	80 (94)	
Conduct problems			
With difficulties	31 (37)	16 (19)	0.004
Without difficulties	54 (63)	69 (81)	
Hyperactivity			
With difficulties	47 (55)	23 (27)	< 0.001
Without difficulties	38 (45)	62 (73)	
Peer relationship problems			
With difficulties	22 (26)	08 (09)	0.007
Without difficulties	63 (74)	77 (91)	
Total difficulties score			
With difficulties	55 (65)	20 (23)	< 0.001
Without difficulties	30 (35)	65 (77)	
Pro-social behavior			
With difficulties	06 (07)	06 (07)	1.000
Without difficulties	79 (93)	79 (93)	

*SDQ* Strengths and Difficulties Questionnaire, *f* frequency, *%* percentage

*p value for McNemar’s test/*p* ≤ 0.05

Significant statistical differences were found when comparing mothers and teachers in regard to the four specific scales of difficulties and total difficulties. Note that the mothers considered their children to present more emotional symptoms, conduct problems, hyperactivity, peer relationship problems, and total difficulties than the teachers. In regard to pro-social behavior, no statistically significant differences were found in regard to the comparisons between mothers and teachers.

In regard to the level of agreement obtained between assessments (mothers and teachers), note that reasonable agreement levels were found for conduct problems (kappa = 0.29 *p* = 0.003) and total behavioral problems (kappa = 0.21; *p* = 0.007), in addition to minimum indexes for hyperactivity (kappa = 0.19; *p* = 0.035).

Similarly, the same differences were found for continuous scores. The means of the mothers were greater than those presented by the teachers for the total difficulties score (mothers: $\overline{x}$ = 17.5; *σ* = 6.98; teachers: $\overline{x}$ = 9.33; *σ* = 7.28; *p* <  0.001) and for the four scales concerning symptoms: emotional symptoms (mothers: $\overline{x}$ = 3.19; *σ* = 2.52; teachers: $\overline{x}$ = 2.26; *σ* = 1.89; *p* <  0.001), conduct problems (mothers: $\overline{x}$ = 3.32; *σ* = 2.56; teachers: $\overline{x}$ = 1.59; *σ* = 2.23; *p* < 0.001), hyperactivity (mothers: $\overline{x}$ = 6.79; *σ* = 2.76; teachers: $\overline{x}$ = 4.04; *σ* = 3.23; *p* < 0.001), and peer relationships (mothers: $\overline{x}$ = 2.20; *σ* = 2.20; teachers: $\overline{x}$ = 1.42; *σ* = 1.90; *p* < 0.001).

### The profiles of families in terms of vulnerability and risk variables

Table 2 presents comparisons concerning social vulnerability, maternal depression, and chronic adversities presented in the family context of children according to their distribution in the three groups.

**Table 2:** Comparisons between the groups regarding social vulnerability, maternal depression, and chronic adversities (*n* = 85)

Groups	G1 (*n* = 18)	G2 (*n* = 39)	G3 (*n* = 28)	Test value	*p* value*
Social vulnerability	*f* (%)	*f* (%)	*f* (%)		
Maternal education					
Up to 8 years	05 (28)	16 (41)	01 (04)	11.961	0.003
More than 8 years	13 (72)	23 (59)	27 (96)		
Paternal education					
Up to 8 years	10 (56)	20 (51)	11 (39)	1.429	0.489
More than 8 years	08 (44)	19 (49)	17 (61)		
Marital status					
Single-parent family	11 (61)	32 (82)	23 (82)	3.589	0.165
Two-parent family	07 (39)	07 (18)	05 (18)		
Socioeconomic class					
C, D, and E	06 (33)	13 (33)	09 (32)	0.012	0.994
A and B	12 (67)	26 (67)	19 (68)		
Family income					
Up to 3 minimum wages	13 (72)	24 (62)	10 (36)	7.044	0.030
More than 3 min. wages	05 (28)	15 (38)	18 (64)		
Government benefit					
Present	04 (22)	12 (31)	02 (07)	5.465	0.065
Absent	14 (78)	27 (69)	26 (93)		
Maternal depression					
Presence	11 (61)	15 (38)	00 (00)	21.376	< 0.001
Absence	07 (39)	24 (62)	28 (100)		
Chronic adversities	M (SD)/Med	M (SD)/Med	M (SD)/Med	Test value	*p* value**
	1.50 (0.92)	1.95 (1.61)	1.04 (1.17)	6.651	0.036
	2.00	2.00	1.00		

Note. G1 = children with behavioral difficulties according to mothers and teachers; G2 = children with behavioral difficulties according to mothers or teachers; G3 = children without behavioral difficulties according to mothers and teachers

*f* frequency, *%* percentage, *M* mean, *SD* standard deviation, *Med* median

*p value for the chi-square test

**p value for the Kruskal-Wallis test/*p* ≤ 0.05

Statistically significant differences were found between G1, G2, and G3 in regard to maternal education, family income, maternal depression, and chronic adversity. The comparison concerning maternal education revealed significant differences between G1 and G3 (*χ*^2^ = 5.660, *p* = 0.017) and between G2 and G3 (*χ*^2^ = 12.075, *p* < 0.001). Significant differences were also found in terms of family income between G1 and G2 (*χ*^2^ = 4.349, *p* < 0.037) and between G1 and G3 (*χ*^2^ = 5.841, *p* = 0.016). No differences were found between groups in terms of paternal education, marital status, socioeconomic status, or receiving governmental financial aid. Considering the variable maternal depression, however, statistically significant differences were found between G1 and G2 (*χ*^2^ = 13.876, *p* < 0.000) and between G1 and G3 (*χ*^2^ = 22.489, *p* < 0.001). G1 was the group in which mothers more frequently presented current symptoms of depression in comparison to the other two groups, while no differences were found between G2 and G3.

Comparisons concerning chronic adversities revealed significant differences between G2 and G3 (*F* = 363.000, *p* = 0.016), but no differences were found between G1 and G2 or between G1 and G3.

### The predictive effect of adverse cumulative variables on child behavior

Table 3 presents the weight of adverse cumulative contextual variables on child behavior, including data concerning coefficients and significance of the adjusted ordinal model.

**Table 3:** Multiple ordinal regression with contextual variables for behavioral problems (*n* = 85)

Parameters	Behavioral problems (SDQ—mothers and teachers)					
	Estimate	SE	Wald	df	*p* value	CI 95%
Limit						
Behavior problems =.00	2.590	0.676	14.960	1	< 0.001	− 3.915;− 1.266
Behavior problems = 1.00	0.039	0.579	0.005	1	0.946	− 1.097;1.175
Location						
Maternal depression = 1.00	− 1.955	0.572	11.683	1	< 0.001	− 3.077;− 0.834
Mother schooling = 1.00	− 0.340	0.527	0.417	1	0.518	− 1.374;0.693
Monthly income = 1.00	− 0.513	0.458	1.254	1	0.263	− 1.410;0.384
Chronic adversity	− 0.086	0.157	0.301	1	0.583	− 0.222;0.394

*p* ≤ 0.05

Note. *SDQ* Strengths and Difficulties Questionnaire, *SE* standard error, *df* degrees of freedom, *CI (95%)* confidence interval 95%

Data suggest that the adjusted model is significantly better than the null model [*G*^2^(4) = 24,792, *p* < 0.001). Additionally, the multivariate model was statistically significant [chi-square (58) = 50,367, *p* = 0.752; *D*(58) = 57,402, *p* = 0.497] and showed moderate effect size (R^2^MF = 0.253; R^2^N = 0.288; R^2^CS = 0.139). According to the model, children are more likely to present behavioral problems when their mothers present indicators of depression, according to the assessments of both mothers and teachers (*b* = 1.955, *p* = 0.001).

## Discussion

This study was intended to verify associations between indicators of social vulnerability, chronic adversity, and maternal depression, and the weight of such associations, with behavioral problems among school children, as assessed by their mothers and teachers. In this study, mothers and teachers were considered distinct sources of information, and the information they provided on the children’s behaviors was combined considering the presence or absence of difficulties manifested in the two developmental contexts of family and school. The hypothesis guiding this study that social vulnerabilities, chronic adversity, and maternal depression impact behavioral problems among school children was partially confirmed, as data analysis revealed peculiarities regarding such variables, which deserve to be highlighted.

The assessments of the children’s behavior from the perspectives of mothers and teachers in general showed that mothers identified more behavioral problems in children than did the teachers. This finding is in agreement with those reported in the studies conducted by De Los Reyes et al. (2015) and Martel et al. (2017), which indicate low to moderate agreement among informants. In this same direction, Clark et al. (2017) consider that agreement between assessments of parents, teachers, and children is rarely high, however, emphasizing that varied information enriches the understanding of the associations between academic conditions, personality, psychosocial functioning, behavioral aspects, mental health, and social adjustment of school children. According to the mothers’ assessments, a larger number of children experienced difficulties concerning emotional symptoms, while the teachers identified a larger number of children with externalizing problems expressed through conduct problems and hyperactivity. Such results are similar to those reported by Kovess et al. (2015), who note that externalizing problems are more visible to teachers than internalizing problems.

Analysis of this discrepancy between assessments should take into account that the interaction of mothers and teachers with children occurs in contexts that exhibit different demands, in addition to the fact that observers are guided by different criteria. In the family context, mothers have a more detailed picture of their children’s behavior due to the large range of daily situations, which are not always structured (Leis et al., 2014). In the case of the mothers, the parameter is one specific child. In the classroom, in contrast, teachers have more structured situations to assess children and the teachers’ references include comparing the behavior of a set of children with similar demographic parameters. In this sense, when the assessments of mothers and teachers were combined, we accessed a larger set of information concerning the behavior of children, focusing on aspects of contextual comparisons and individual and collective parameters, as proposed by Miller et al. (2014) and De Los Reyes et al. (2015).

The literature has recognized the relevance of assessments performed by teachers; however, few studies address behavioral difficulties of children using multiple informants and combined data as a strategy to identify the presence of problems in more than one context of life. The predominance of the mother as the only informant may compromise the results of assessments, especially when a mother presents a psychopathological disorder (Leis et al., 2014), such as depression. Such a disorder may influence the individual’s perception of child behavior, and avoiding this influence justifies the use of distinct and combined sources of information. Therefore, we note that one of the contributions of this study, in addition to including multiple informants, is the combined analysis of children’s behavioral outcomes, which enabled verifying problems in two contexts, family and school, to estimate how many children face these sorts of difficulties, information that is relevant for practices in the mental health field.

Another aspect to be analyzed involves social vulnerability, which was assessed considering different social and economic factors, among which are low maternal educational level and income. These are relevant social determinants associated with the presence of behavioral problems among children, according to the assessments by mothers and/or children, indicating aspects to be considered when planning preventive practices. Note that these findings are consistent with those reported by Correia et al. (2014), who identified association between child behavioral problems and low socioeconomic status and low maternal educational level, indicating a potential profile of cumulative vulnerability favoring behavioral problems among children. Families with low socioeconomic status generally have high rates of divorce, unemployment, and a larger number of members, while parents with a high socioeconomic level have a higher educational level and invest more in their children’s education (Carneiro, Meghir, & Parey, 2013; Piccolo et al., 2012).

The associations between mental health conditions and vulnerability indicators have been widely recognized by the World Health Organization (WHO, 2017a, 2017b), which highlights low schooling, lower income, worse material and economic conditions, and less social support, as possible determinants that negatively influence health mental health of adults and children, favoring the accumulation of vulnerability and risk conditions. This developmental scenario focuses on the relevance of the present study, which encompasses diverse and competing contextual variables that influence children’s developmental outcomes in the perception of different informants.

The presence of current depressive symptoms among the mothers was associated with behavioral problems among the children, as indicated by the mothers and/or teachers, characterizing problems in two contexts, family and school. Such an association was also verified by Leis et al. (2014) and Conners-Burrow et al. (2016), who noted an increase in behavioral problems among children who had early experience with maternal depression. In this sense, when we considered the behavior of children from the perspectives of mothers and teachers together, we verified that, regardless of the informant, children living with maternal depression more frequently experienced behavioral problems, including in the school context, characterizing the need for specific mental health practices directed to this group, which was identified as the most vulnerable.

The presence of chronic adversities was also verified to identify variables with a potential negative impact on school-aged children. This study reveals that children facing behavioral difficulties, according to the combined assessments of mothers and teachers, lived in family environments that presented more chronic adversities, indicating cumulative and recurrent adversity in these children’s contexts of life. These findings corroborate the study conducted by Hildebrand et al. (2015), who identified an association of two or more risk factors for more than half of the sample under study.

The identification of differences among groups, especially for children facing problems in the family and school contexts (G1) in regard to social vulnerability, current maternal depression, and chronic adversity, characterizes a group that requires greater attention, as it is exposed to multiple risks. This information highlights the relevance of investigating the presence of cumulative risk in the family context to understand developmental outcomes among children (Evans et al., 2013; Goodman et al., 2011).

In regard to the identification of the predictive effect of cumulative risk variables and vulnerability, as potential predictors of behavioral problems among children, only maternal depression appears as an explanatory variable for the presence of behavioral problems among children in the context of multiple adverse conditions. These findings are in agreement with Bagner et al. (2010), who stress that living with maternal depression increases a child’s likelihood of presenting externalizing and internalizing behavioral problems up to the age of 12 years old. Therefore, maternal depression was the only adverse condition with the power to predict the behavioral problem outcome, confirming the relevance of considering such a variable when addressing child behavior, especially considering the high prevalence of depression among women of childbearing age (World Health Organization [WHO], 2017a, 2017b).

As the positive aspects of this study, we highlight the presence of multiple informants, the methodological care adopted in the systematic assessment of the participants, and the use of validated instruments, in addition to the inclusion of diverse variables to identify, in the same sample, vulnerability indicators that potentially impact the behavior of school children. It is highlighted as the main strength of the study the inclusion of children in the groups considering the presence or absence of behavioral problems in the two main development contexts for the school period, namely, family and school, thus highlighting relevant variables associated with vulnerability and to developmental resources in both contexts, which may favor preventive care and target groups with potential risks.

This study’s limitations include the sample size, lack of a homogeneous distribution between groups in regard to the sex of children, and the identification of depressive symptoms using a screening instrument, which limit the generalization of results. Further studies adopting longitudinal designs, considering the influence of contextual risks over the course of a child’s development, including other sources of information, in addition to the reports of mothers, are needed, as well as observational measures. The relevance of inclusion in new studies of parents’/stepfathers’ evaluations, as well as studies that address the characteristics of the various family configurations in which children are inserted as conditions that can influence the behavior of the school-aged children, is also highlighted. Another relevant point to be considered in new studies is the inclusion of variables that may function as protective factors, which in a cumulative way to vulnerability and risk conditions may favor a more complete and complex analysis of the mechanisms that favor or hinder children’s behavioral problems.

## Conclusions

In this study, low maternal educational level, low family income, the presence of more chronic adversity, and living with current maternal depression are factors associated with the outcome of behavioral problems among children in both family and school contexts, showing the importance of including such factors in assessment protocols intended to address the mental health of school-aged children. Note, however, that among these indicators, current maternal depression emerged as the most relevant variable in comparison to the remaining adversities analyzed here. Therefore, this condition requires specific care when implementing mental health actions.

Finally, these results can contribute to and have implications for the planning of mental health programs, confirming the relevance of identifying maternal depressive symptoms and multiple adversities, including social vulnerability indicators as conditions or events that demand attention.
